# Intelligent Voice System Design for Optimizing E-Business Advertising Rhetoric Based on SVM Algorithm

**DOI:** 10.1155/2022/1944275

**Published:** 2022-10-08

**Authors:** Chunfeng Guo

**Affiliations:** Department of Information Engineering, Shandong Foreign Trade Vocational College, Qingdao, Shandong 266100, China

## Abstract

With the emergence and development of artificial intelligence, design is no longer a process that can be completed only by creativity and knowledge. This article introduces research based on voice data mining and further discusses the optimization and application of rhetorical methods for e-commerce advertising. This article is working from two main aspects: one is to use association rule models in speech data mining to obtain useful rule relationships between Chinese prosody parameters, and the other is to use neural networks and data items in speech data mining. Using computer technology can make online advertisements interact with users. In addition to clicking on advertisements to jump to product pages, online advertisements also have multichannel sensory feelings, which can provide rich information and stimulate interest through interaction with users. We need to optimize the rhetoric of the advertisement and pay attention to the combination of the beauty of speech and the content of the advertisement when designing the advertisement. At the same time, we must also pay attention to the accuracy and elegance of the advertising language and wording.

## 1. Introduction

In this article, we first understand the mapping relationship between the basic frequency of two words and the syllable prosody rule through the neural network method, and then, use the network obtained after training to calculate the required frequency, and finally, use the calculated frequency with Yu synthesis [1]. Throughout the experiment, we obtained a better learning effect, and at the same time, the result of using the neural network to generate the basic frequency change is in full compliance with the certified tone change law. To understand the prosodic changes of the syllables in a sentence, first use cluster analysis to obtain the basic frequency patterns of common syllables in the sentence [2]. These basic frequency patterns can perfectly match today's common tone curves. Based on the basic frequency model, we convert the basic frequency of the training data into a high-level description, and use various speech data mining methods to learn the prosody rules and obtain good experimental results [3]. The research experiments in this paper show that it is feasible and effective to use speech data mining technology to extract and learn prosody rules in speech synthesis. In the future research of computer-aided intelligent generative design systems, language can combine more design principles with advanced technologies such as computer graphics and artificial intelligence to continuously improve the system [4].

Based on the research of voice data mining, we discussed in detail the dissemination and design methods of e-commerce advertisements [5]. The Internet e-commerce banner advertisement design system in the article is structured and can collect a large number of design processes and codes. Through mining processes and codes, artificial intelligence technology is used to improve the level of system automation and intelligence [6]. Many websites make use of embedded e-commerce advertisements to obtain corresponding income. Therefore, we will not only see e-commerce advertisements on e-commerce sites but also on non-e-commerce sites, so the scope of dissemination is very wide. When the spread of advertisements is guaranteed by people, and how advertisements can arouse people's attention has become a more important link at present [7].

Based on this question, we will introduce the concept of graphic rhetoric. Graphic rhetoric has become an important tool for e-commerce advertising to create ideas through literary rhetoric theory and practical results and interpret it into graphic design [8]. The common rhetoric method used to promote graphic creativity includes: metaphor, symbolism, exaggeration, contrast, personification, and other rhetoric methods. Explore the rhetoric used in each creative method and analyze its characteristics and meaning. Deduct more graphic rhetoric types and methods and learn more graphic rhetoric methods to grasp the process of graphic rhetoric, and improve the performance of e-commerce advertising graphics by continuously optimizing graphic rhetoric methods [9].

## 2. Related Work

Some research introduces the application of parametric design and shape grammar in the field of e-commerce banner advertisement design, and based on these principles, initially constructs a complex banner advertisement auxiliary design system [10]. This describes the system framework, implementation technology, and related principles. The literature introduces concise, appropriate, and easy-to-master advertising language features, and respectively introduces the rhetorical beauty of language and the application of content beauty in actual advertising [11]. Some research introduces the model of the mobile communication network data analysis system and the technical principles corresponding to each module. Key issues in mobile networks: Volte's sound quality issues have been studied [12]. The literature introduces the development status of the digital advertising industry and the new characteristics of brand communication in the context of digital advertising. It believes that lack of transparency, brand security threats, and low advertising awareness are the main problems that cause brand communication [13]. In response to these problems, we have proposed more effective measures to promote the development of digital advertising, such as establishing a transparent brand communication environment, strengthening the control of the advertising environment, and integrating visibility verification standards to improve the reliability of advertisers [14]. Some research introduces the process of extracting relevant rules for data mining and introduces the prosodic rules for speech synthesis [15]. We will improve the Apriori algorithm in the extraction process to obtain the HLA Apriori algorithm suitable for the speech itself, which is used in the learning of the syllable prosody changes of ordinary sentences.

## 3. Voice Data Mining and Artificial Intelligence Model Design

### 3.1. Speech Feature Extraction

MFCC was proposed by Stevens, Volkman, and Newman in 1937. The listener judges a perceptual scale at the same distance from each other. The Mel is a measurement unit of perceived pitch or tone frequency. By equating a 1000 Hz tone with a listener threshold of 40 dB, a 1,000 Mel tone, the reference point between this scale and the normal frequency measurement, can be defined. At about 500 Hz, the listener will determine larger and larger intervals and produce the same pitch increment. Finally, 4 octaves above 500 Hz on the Hertz scale are judged to contain about 2 octaves on the scale. The Mel scale is almost linear below 1 kHz, and counts above. The following formula is used to calculate the Hertz (Hz) for a given frequency *f*. MFCC has a strong noise suppression function, and the relationship between frequency and actual frequency *f* is as follows:

(1) $$\begin{array}{c} \text{Mel}\left(f\right)=2595lg\left(1+\frac{f}{700}\right) \end{array}$$

**Figure 1:**
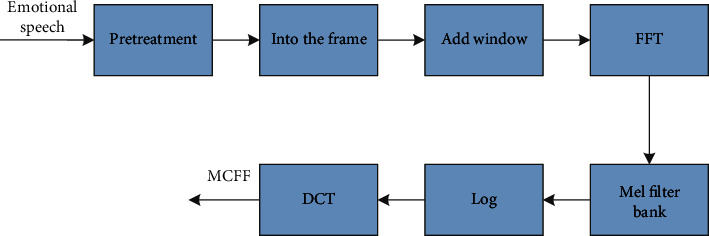
MFCC feature extraction process.

MFCC feature extraction process is as shown in Figure 1.

1. The preprocessing, framing, Windows, FFT, and Mel filter bank, and frequency packing processes of MEDC feature extraction are the same as those of MFCC feature extraction.
2. The average logarithm of the energy of each filter in the process of calculating the average logarithm of chuwihagoi energy. The average value represents the energy of each filter in the filter bank.
3. Calculate the difference between the first and second, and combine the first and second difference of filter energy to obtain the dynamic coefficient of the final Mel energy spectrum.

### 3.2. Speech Data Training Classification

First, SVM is used to classify and associate multiple category labels for some commonly used classifications in e-commerce advertising rhetoric. (e.g., painter, happiness, sadness, neutrality, and fear).

**Figure 2:**
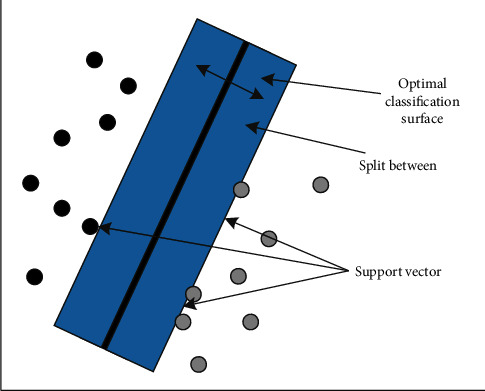
Two classifications principle of support vector machine.

Train the SVM according to the displayed function. The SVM kernel function is used in the SVM training course. In the face of multiple e-commerce advertising rhetoric classifications, RBF is used and the relevant training set data is bound, to deal with the nonlinear relationship between labels and their related attributes. Compared with polynomial kernels, RBF kernels are not so difficult numerically. SVM classification results are provided in matrix table format. The confusion matrix shows the accurate classification and the classification percentage for the specified category. The principle of two classifications is shown in Figure 2.

Assume that the training sample data used for speech emotion recognition is as follows: {*xi*, *yi*}, *xi* ∈ *Rn*, *i* = 1, 2,…, *n*, *x*, emotion recognition feature vector, *y* is the emotion category. As explained below, SVM nonlinear uses the mapping Φ(*x*) to create a linear optimal classification and maps the training set to the nonlinear problem in the dimensional space.

(2) $$\begin{array}{c} y=\omega^{T}\Phi \left(x\right)+b\text{.} \end{array}$$

Need to find the best *ω* and *b*. Formula (2) cannot be solved directly, it is necessary to find the best classification surface. Therefore, the relaxation factor *ξi* is introduced, namely,

(3) $$\begin{array}{c} \min\;J\left(\omega ,\xi \right)=\frac{1}{2}{\left\|\omega \right\|}^{2}+C\overset{n}{\underset{i=1}{\sum }}\xi_{i}\text{,}\\ \;s.t.y_{i}\left(\omega \cdot \Phi \left(x_{i}\right)+b\right)⩾1-\xi_{i}\text{,}\\ \xi_{i}⩾0i=1,2,\cdots ,n\text{.} \end{array}$$

Introduce Lagrange multiplier to transform equation (3)

(4) $$\begin{array}{c} \max\;W\left(\alpha \right)=\;|\;\overset{l}{\underset{i=1}{\sum }}\alpha_{i}-\frac{1}{2}\overset{l}{\underset{i=1}{\sum }}\alpha_{i}\alpha y_{i}x_{i}\cdot x_{j}\text{,}\\ s.t.\;\left\{\begin{array}{c} \overset{l}{\underset{i=1}{\sum }}\alpha_{i}y_{i}=0,\\ C⩾\alpha_{i}⩾0i=1,2,\cdots ,l. \end{array}\right. \end{array}$$

The calculation formula of the weight vector *ω* is

(5) $$\begin{array}{c} \omega =\sum \alpha_{i}y_{i}\Phi \left(x_{i}\right)\cdot \Phi \left(x\right)\text{.} \end{array}$$

The decision function can be expressed as follows:

(6) $$\begin{array}{c} f\left(x\right)=\text{sgn}\left(\alpha_{i}y_{i}\Phi \left(x_{i}\right)\cdot \Phi \left(x_{j}\right)+b\right)\text{.} \end{array}$$

In order to reduce the computational complexity, the kernel function *k*(*x*, *x*_*i*_) is introduced to replace the internal ridge Φ(*x*_*i*_) · Φ(*x*_*j*_). Change (6) to:

(7) $$\begin{array}{c} f\left(x\right)=\text{sgn}\left(\alpha_{i}y_{i}k\left(x,x_{i}\right)+b\right)\text{,} \end{array}$$

Because RBF has good versatility, the kernel function RBF is used as the kernel function of the support vector machine. The formula (6) becomes:

(8) $$\begin{array}{c} f\left(x\right)=\text{sgn}\left(\alpha_{i}y_{i}\exp\;\left(-\frac{\left\|x-x_{i}\right\|}{2\sigma^{2}}\right)+b\right)\text{.} \end{array}$$

### 3.3. Speech Data Recognition Algorithm

(1) The input layer, hidden layer, and output layer assign values between (−1, 1) for the connection weight between neurons and neuron thresholds and specify the learning coefficient and activation function of the neuron. Usually select the effective function to reflect the following: the Sigmoid function or hyperbolic tangent function of the nonlinear characteristics of biological neurons, and the BP algorithm requires conditions that can distinguish effective functions. These two functions are expressed as follows:
Sigmoid function:
  (9) $$\begin{array}{c} f\left(x\right)=\frac{1}{1+\exp\;\left(-x\right)}\text{.} \end{array}$$
Hyperbolic tangent function:
  (10) $$\begin{array}{c} f\left(x\right)=\tanh\;\left(x\right)=\frac{e^{x}-e^{-x}}{e^{x}+e^{-x}}\text{.} \end{array}$$
(2) Randomly select *X*_*k*_ = (*x*_1*k*_, *x*_2*k*_,…, *x*_*mk*_), *Z*_*k*_ = (*z*_1*k*_, *z*_2*k*_,…, *z*_*qk*_) and send it to the network.
(3) Use the network settings to calculate the output *bj* of each neuron in the hidden layer.
  (11) $$\begin{array}{c} \left\{\begin{array}{c} s_{j}=\overset{m}{\underset{i=1}{\sum }}w_{ij}x_{i}-\theta_{j}\text{,}\\ y_{j}=f\left(s_{j}\right)\text{.} \end{array}\right. \end{array}$$
(4) Use the network settings to calculate the response *C*_1_ of the neurons in the output layer.
  (12) $$\begin{array}{c} \left\{\begin{array}{c} u_{l}=\overset{p}{\underset{j=1}{\sum }}v_{jl}y_{j}-\gamma_{l}\text{,}\\ C_{l}=f\left(u_{l}\right)\text{.} \end{array}\right. \end{array}$$
(5) Use specific data to calculate and output the generalized error *d*_1_^*k*^ of the neurons in the layer.
  (13) $$\begin{array}{c} d_{t}^{k}=\left(z_{l}^{k}-C_{l}^{k}\right)f^{\prime }\left(u_{l}\right)d_{t}^{k}=\left(z_{l}^{k}-C_{l}^{k}\right)f^{\prime }\left(u_{l}\right)\text{.} \end{array}$$
(6) Calculate the generalized error *e*_*j*_^*k*^ of each neuron in the hidden layer:
  (14) $$\begin{array}{c} e_{j}^{k}=\left[\overset{q}{\underset{l=1}{\sum }}v_{jl}d_{l}^{k}\right]f^{\prime }\left(s_{j}^{k}\right)\text{.} \end{array}$$
(7) Randomly select other input-output data sets, and then, return to (3) for learning. Train all data sets. This is the process in which the network uses the sample set to complete the learning process.
(8) Repeat the next learning process until the global error of the network is less than the set value or the number of training times reaches the set number.
(9) Perform performance tests on trained networks to ensure that they meet the requirements.

In the above process, (3) and (4) are forward processes, and (5) to (7) are backward propagation processes. The neural network, finally, converges to the weight value through multiple training and modification.

The corpus saves sampling data in WAVE file format and provides a word index table. Some index tables provided by the corpus are listed in Table 1.

### 3.4. Voice Data Mining Model

The sample points are classified and adjusted based on the *K*-means method. When the clustering results are the same or reach the specified number of times, the clustering ends, otherwise the following adjustments are made:

#### 3.4.1. Delete

If the number of sampling points of a specific type is less than MinS, delete this type, and the sampling points of this type will no longer participate in future calculations.

#### 3.4.2. Decomposition

Assuming that *m* classes are generated through multiple iterations, one of the *n* features of each sample class requires the maximum root mean square error. Take the average of the maximum root mean square deviations of all types and record it as $\bar{S_{\max}}$.

(15) $$\begin{array}{c} S_{\text{threshold}}=\bar{S_{\max}}∙\frac{J}{1+e^{-\left(m-C\right)}}\text{.} \end{array}$$

#### 3.4.3. Combination

Suppose that *m* classes are generated through multiple iterations, then, calculate the minimum value of each condensation point type of the other condensation points and average these minimum values as follows. The average value is recorded as $\bar{D_{\min}}$. After syllable division and period marking are performed on the training sentence waveform, length normalization, and moving average can be used to obtain a uniform length and a smooth basic frequency sequence, so that the original data can be summarized. The calculation formula is shown in the formula.

(16) $$\begin{array}{c} \text{Dist}\left(A,B\right)=\sqrt{\overset{n}{\underset{i=1}{\sum }}{\left(\left(a_{i}-\bar{a}\right)-\left(b_{i}-\bar{b}\right)\right)}^{2}}+\left|\bar{a}-\overset{⟶}{b}\right|\text{,} \end{array}$$

In the formula $A=\left(a_{1},a_{2},\ldots a_{n}\right),\bar{a}=1/n\sum_{i=1}^{n}a_{i},B=\left(b_{1},b_{2},\ldots ,b_{n}\right)\bar{b}=1/n\sum_{i=1}^{n}b_{i}\text{.}$ The length of the basic frequency sequence of each syllable is normalized and the moving average is performed, the average value of each basic frequency sequence is calculated, and the zero-average processing is performed, and the zero-average basic frequency mode is finally obtained. The calculation formula is

(17) $$\begin{array}{c} \text{Dist}\left(A,B\right)=\sqrt{\overset{n}{\underset{i=1}{\sum }}{\left(a_{i}-b_{i}\right)}^{2}}\text{.} \end{array}$$

### 3.5. Algorithm Simulation

Create a voice emotion database, which contains emotional dialogues from a variety of movies. Initially, all files were cut into.mp3 format, converted to.Wav file format, and Berlin sentiment database was used. It contains 406 audio files for 5 emotion categories. The emotional categories of anger, sadness, happiness, neutrality, and fear are 124, 59, 68, 75, and 69 words, respectively. Use the RBF function to train the MFCC and MEDC feature vectors of LIBSVM, and use LIBSVM to test these feature vectors. Run the experiment while changing the cost of the RBF kernel, and experiment with a separate file. SVM classification results are provided in matrix table format. The confusion matrix shows the exact classification and classification percentage of the specified category.

In Table 2 shows that the SVM implemented by the confusion matrix is used in Chinese speech emotion. The feeling of anger can reach 89.36%, and the recognition rate of happiness can reach 90.6%. The biggest classification is found in sadness and fear. The overall emotion recognition rate in China is 83.55%.

In Table 3 shows the SVM confusion matrix implemented in speech emotion using a variety of one-to-one classification methods. Observations show that the biggest categories are happiness and fear. Due to fear, the highest anger can reach 88.26%, while the lowest recognition rate is 44.23%. The overall recognition accuracy of speech emotion is 57.91%.

**Figure 3:**
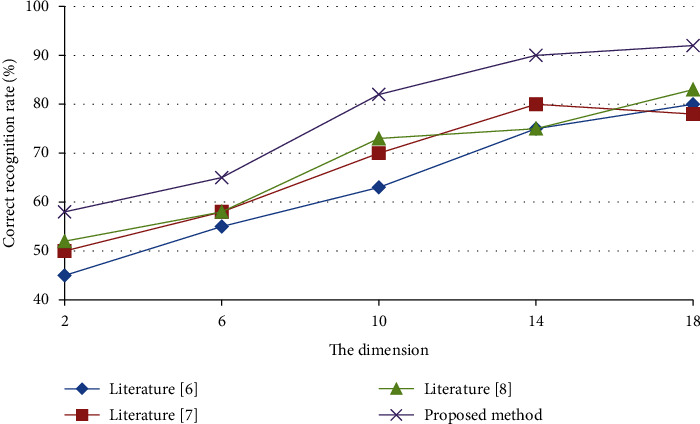
Comparison of speech emotion recognition results obtained by algorithms in different dimensions.

In Figure 3 shows the speech emotion recognition results of each dimension obtained by using four dimensionality reduction methods.

**Figure 4:**
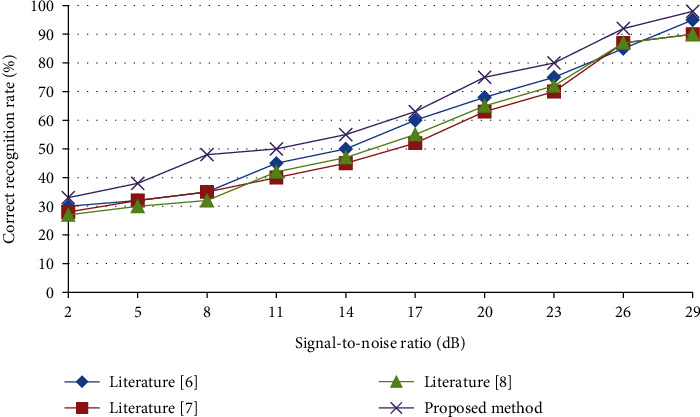
Comparison of speech emotion recognition results obtained by several algorithms under different signal-to-noise ratios.

In Figure 4 shows the comparison of speech emotion recognition results obtained by multiple algorithms under various signal-to-noise ratios. It can be seen that the method proposed in this paper achieves the highest recognition accuracy with different dimensionality reductions and different signal-to-noise ratios.

## 4. Optimization of E-Commerce Advertising Rhetoric Methods

### 4.1. Rhetorical Characteristics of E-Commerce Advertising

The Internet was born in 1980. With the rapid development of computers and new media technologies, Internet advertising has become the most important part of the advertising industry since its early development and has become an indispensable part of advertising research. Due to the popularity of the Internet, Internet advertising continues to affect everyone's daily life. “Internet advertising is a new form of advertising. The purpose is to use various forms of the Internet to communicate and persuade identifiable investors. Internet advertising is a new type of advertising with multiple forms. The main method of communication is through the Internet. Advertising service providers and delivery platforms insert specific Internet advertisements in Web documents. Therefore, Internet advertisements, mainly, include static images and dynamic advertisements, as well as photos, videos, and interactive animations. Compared with the characteristics of advertisements, Internet advertisements also inherit some unique features include:

1. *Iterative Updating Is Fast and Timeliness Is Strong*. Internet advertising is quickly repeated and coordinated based on market feedback and marketing plans, because feedback data from various markets can be quickly obtained from the Internet, and the data is updated very quickly, the iterative process is relatively fast and the cost is low. Therefore, compared with existing media advertising, Internet advertising is more time-sensitive.
2. *Fast Spreading Speed and Wide Application Range*. With the continuous advancement of Internet technology, all customers can receive new advertising content at the same time, and the Internet speed will be faster, tariffs will be reduced, advertisements will load faster, and websites in various fields will insert Internet advertisements. Internet advertising will spread widely.
3. *Strong Interaction*. The use of computer technology enables users of online advertisements to interact with web pages or applications. The continuous development of web development technology, especially the birth of touch screen interaction technology, will enable advertisements distributed on the Internet to better interact with users. In addition to clicking on the ad to jump to the product page, online advertising also has a multichannel sensory experience, which can provide a wealth of information and stimulate greater user interest through interaction with users.

### 4.2. Calculation of Rhetorical Complexity of Advertising

Complexity is the description of the internal characteristics of an objectively existing thing or event, that is, the complexity of the event. The qualitative and quantitative interpretation of image complexity helps to reflect the image complexity and the ability to perform certain tasks in the image processing process. For example, image edge detection, automatic image annotation and recognition, image improvement, and target image extraction.

First, we will introduce the complexity of the generalized set, and then perform a mathematical quantitative analysis of the complexity to obtain the complexity of the internal state, which is calculated as follows:

(18) $$\begin{array}{c} C=-\overset{k}{\underset{i=1}{\sum }}n_{i}\cdot \log\left(\frac{n_{i}}{N}\right)\text{.} \end{array}$$

It can be understood from the formula that the larger the generalized set, the greater the individual difference and the greater the value of complexity C. The complexity of the image can also be calculated using formulas. When using this method to calculate the complexity of an image, the grayscale histogram of the image should be considered, that is, the calculation of information.

(19) $$\begin{array}{c} H=-\frac{\overset{k}{\underset{i=1}{\sum }}n_{i}}{N}\cdot \log\left(\frac{n_{i}}{N}\right)=\frac{C}{N}\text{.} \end{array}$$

Using gray to calculate the image complexity can not only reflect the distribution of gray but also quantitatively analyze the symmetry and reproducibility of the image. You can also use this method to calculate the image complexity to obtain the image complexity.

The graphic advertisement photos of the e-commerce scene described in this article are composed of multiple layers, and each layer can perform complex image processing, especially for the background image gradient, blur, texture, and style of the advertisement. Research through graphic advertising design elements and many aspects of the design process to design and create complex graphic advertising images.

Internet slogan advertisements on e-commerce websites are mainly published on e-commerce websites in the form of advertisements, and their main purpose is to carry out marketing activities and product promotions for member stores. E-commerce banner ads not only have the characteristics of advertising but also have some general characteristics, such as:

#### 4.2.1. Wide Spread and Clear Purpose

Because many websites insert e-commerce advertisements to obtain revenue, e-commerce advertisements will be posted on various non-e-commerce web pages, not just e-commerce websites. The recommendation algorithm optimizes e-commerce advertisements to support high-end advertisements that can be personal and highly targeted.

#### 4.2.2. The Language of the Copy Content is Popular

The language and text of e-commerce advertisement copy content should be concise and clear, because e-commerce print advertisements are more popular, users are not interested in them, and they spend less time on the Internet.

### 4.3. Key Technologies for E-Commerce Advertising Optimization

The system toolset contains four tool libraries that can be used to create layouts, texture tools, color tools, and shapes in the current form. The following is a detailed introduction of each tool library. Shape layout is a tool that can transform and arrange the input or generated shapes according to the default background. Depending on the shape and size, other layout methods are used. The shape layout tool has two layout methods: a layout template created based on the syntax of shapes; a layout based on image learning with tags; and a layout template used for probability estimation. The rules of the layout can be adjusted according to the design of the parameters. You can also adjust the parameters to adjust the placement plan appropriately.

Probability estimation model layout: due to the limitations of the use of layout graphics syntax and the difficulty of being more specific in the layout method, these two methods are used for different types of e-commerce banner ads. This method uses a well-trained neural network to recognize the copied content of the advertisement image and the product image, divide the two parts into other areas, and classify the recognition and division results and the layout area of the graphics.

The texture tool mainly processes the entire image, including three types of tools: blur, texture overlay, and style.

#### 4.3.1. Blur Tool

The blurred background is one of the important image processing processes to create the default background, because the blurred background is a common default background in the process of creating the basic background of graphics. The blur tool has two main functions. One is to perform Gaussian processing on the entire photo, and the other is to create blurred images of color patches. The image blur (Gauss) function uses the ImageFilter module of Python's image processing library PIL (Python Imaging Ligrary) to blur the image. Gaussian blur (also known as Gaussian smoothing) uses a Gaussian function to blur the image. The purpose is to reduce the noise in the image and reduce the details.

**Figure 5:**
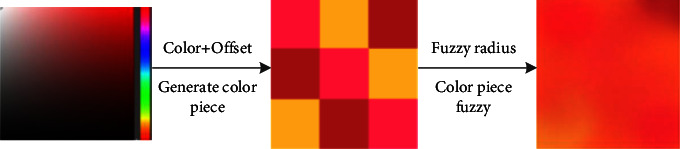
Schematic diagram of the color block blur image generation process.

Another function of the blur tool is to create blurred images of color blocks. After generating color blocks with a specific color and color gamut range specified by the user, this function will generate a default background image with blur (Gaussian). The flowchart is shown in Figure 5.

#### 4.3.2. Texture Overlay

Texture overlay requires two photos, namely, the original image and the texture material. Process the texture material and apply the texture to the original image to produce the result of the texture image.

### 4.4. E-Commerce Advertising Communication Path

#### 4.4.1. Oppose Digital Fraud and Increase the Transparency of Traffic Monitoring

Traffic fraud has become a stubborn disease that plagues the global digital advertising industry. Although it cannot be eradicated, it can be controlled. At present, China's digital advertising technology is very mature, and there is no technical problem preventing fraud, but there is a lack of industry consensus. Joint fraud prevention measures in the industry are crucial because digital fraud seriously damages the overall interests of all parties involved in the advertising transaction link.

The first is to strengthen industrial control, improve industrial regulations, curb false traffic and digital fraud, and actively promote the construction of a healthy and transparent digital ecological environment. Second, a third-party independent monitoring agency needs to be deployed to integrate digital advertising monitoring standards and improve the transparency of traffic monitoring.

#### 4.4.2. Open Data Sharing and Increase the Transparency of Data Circulation

In the context of big data, the accumulation of big data enables the advertising industry to systematically study the characteristics of the target population and achieve accurate digital distribution. Data fragmentation and data barriers are the biggest obstacles to the innovation and development of digital advertising. The biggest obstacle to sharing opening data is the uneven distribution of benefits among all parties in the digital supply chain. Since digital advertising belongs to an emerging industry and various systems are not yet complete, blindly open data may bring security risks. Therefore, an integrated data trading platform must be established, and a scientific data circulation system must be established so that media stakeholders can release data and improve the transparency of data circulation, which will help us fully integrate data.

To increase the display rate of advertisements, participants need to work together in all aspects of digital advertising. For advertisements that have not yet attracted us, the brand still needs to develop a strategy to convey the message to relevant consumers. Ads in apps, native ads, and ads that are more closely integrated with media operator content are not easy to block. Brands must have confidence to make consumers willing to accept brand messages, develop content strategies, sponsor and establish partnerships, and so on. Advertisers are victims of the opaque digital advertising industry chain and are often organizers. Advertisers usually over-buy certain performance indicators and ignore the quality of advertising traffic, which indirectly leads to the generation of invalid traffic. Therefore, the establishment of a scientific and reasonable evaluation method for advertisers can effectively reduce the digital fraud motives of agencies and media and improve the display effect of advertisements.

## 5. Conclusions

The framework of this article starts from the actual Chinese pronunciation, learns more complex prosody rules, and uses data mining technology to improve the quality of synthesized speech and improve the synthesized speech, making the synthesized speech more natural. This article summarizes and analyzes related issues in the fields of data mining and speech synthesis and proposes how to use data mining to extract prosody rules and obtain good results. Finally, in the context of e-commerce banner advertising design, based on voice data mining and artificial intelligence, a complex banner advertising picture-aided design system based on parameter design is constructed. The purpose is to enable creativity to be embodied in a visible way and to change the parameters of the design process to create different design systems. More importantly, through the design process of many Internet banner advertisements, more relevant design thinking and design habits can be discovered.

## Figures and Tables

**Table 1:** Part of the index table provided by the corpus.

Table name	Content
Word l.dbf	1268 monosyllable information table
Word2.dbf	640 two-character word information table
Word3.dbf	2048 three-character word information table
Word4.dbf	2048 four-character word information table
Erhua.dbf	225 voice information tables
Qing.dbf	349 soft-voiced words information table
Duihuaw.dbf	Dialogue information sheet involving 52 topics

**Table 2:** The SVM implemented by the confusion matrix is used for the recognition result of Chinese speech emotion.

Emotion	Recognition accuracy (%)				
	Anger	Sadness	Happiness	Neutral	Fear
Anger	89.36	0.00	0.00	0.00	9.52
Sadness	4.48	65.42	7.68	5.10	16.56
Happiness	1.28	0.00	90.6	6.52	1.12
Neutral	1.65	8.78	1.25	88.38	0.00
Fear	2.26	25.79	0.00	0.00	71.95

**Table 3:** Recognition results of SVM implemented on German speech emotion using one-to-one multi-class method.

Emotion	Recognition accuracy (%)				
	Anger	Sadness	Happiness	Neutral	Fear
Anger	88.26	0.00	7.86	0.00	3.95
Sadness	0.00	64.00	0.00	36.00	0.00
Happiness	39.63	0.00	42.82	10.71	17.21
Neutral	3.12	43.72	0.00	50.00	3.12
Fear	22.22	14.76	7.42	11.32	44.23

## Data Availability

The data used to support the findings of this study are available from the corresponding author upon request.
